# Protein Arginine Methyltransferase 1 Ablation in Motor Neurons Causes Mitochondrial Dysfunction Leading to Age-related Motor Neuron Degeneration with Muscle Loss

**DOI:** 10.34133/research.0158

**Published:** 2023-06-19

**Authors:** Hyun-Kyung So, Hyebeen Kim, Jinwoo Lee, Chang-Lim You, Chae-Eun Yun, Hyeon-Ju Jeong, Eun-Ju Jin, Yunju Jo, Dongryeol Ryu, Gyu-Un Bae, Jong-Sun Kang

**Affiliations:** ^1^ Department of Molecular Cell Biology, Sungkyunkwan University School of Medicine, Suwon, Korea.; ^2^ Single Cell Network Research Center, Sungkyunkwan University School of Medicine, Suwon, Korea.; ^3^ Research Institute of Aging-Related Diseases, AniMusCure, Inc., Suwon, Korea.; ^4^ Drug Information Research Institute, Muscle Physiome Research Center, College of Pharmacy, Sookmyung Women’s University, Seoul, Korea.

## Abstract

Neuromuscular dysfunction is tightly associated with muscle wasting that occurs with age or due to degenerative diseases. However, the molecular mechanisms underlying neuromuscular dysfunction are currently unclear. Recent studies have proposed important roles of Protein arginine methyltransferase 1 (Prmt1) in muscle stem cell function and muscle maintenance. In the current study, we set out to determine the role of Prmt1 in neuromuscular function by generating mice with motor neuron-specific ablation of Prmt1 (mnKO) using Hb9-Cre. mnKO exhibited age-related motor neuron degeneration and neuromuscular dysfunction leading to premature muscle loss and lethality. Prmt1 deficiency also impaired motor function recovery and muscle reinnervation after sciatic nerve injury. The transcriptome analysis of aged mnKO lumbar spinal cords revealed alterations in genes related to inflammation, cell death, oxidative stress, and mitochondria. Consistently, mnKO lumbar spinal cords of sciatic nerve injury model or aged mice exhibited elevated cellular stress response in motor neurons. Furthermore, Prmt1 inhibition in motor neurons elicited mitochondrial dysfunction. Our findings demonstrate that Prmt1 ablation in motor neurons causes age-related motor neuron degeneration attributing to muscle loss. Thus, Prmt1 is a potential target for the prevention or intervention of sarcopenia and neuromuscular dysfunction related to aging.

## Introduction

Sarcopenia is a progressive loss of skeletal muscle mass and function with age and is recognized as a critical risk factor for frailty and mortality in the elderly [1,2]. The neural input to muscle through neuromuscular junction (NMJ) is a key factor to control muscle contractility and growth [3,4]. The reduction in functional motor units due to impaired nerve terminals, fragmentation of NMJ, decreased neurotransmitter release, and reduction in reinnervation capacity post motor neuron injury or loss has been suggested to contribute to sarcopenia or other neuromuscular pathologies, including amyotrophic lateral sclerosis (ALS) [2,5–9]. The importance of the neuromuscular interaction for muscle maintenance has been shown by the experimental result reporting that the loss of motor input due to denervation can cause a drastic muscle atrophy with up to 80% loss of muscle fiber size and the retrograde signaling from muscle fibers is essential for the maintenance of motor neurons [10]. However, the underlying mechanisms of age-related loss of motor units and neuromuscular dysfunction are not clearly defined. Cellular stress pathways, such as oxidative stress, chronic inflammation, and mitochondrial dysfunction, have been implicated in motor neuron degeneration [11–16]. Some of the genetic factors that have been discovered to cause cellular stress in motor neurons include mutations in genes encoding RNA binding proteins (fused in sarcoma [FUS] and TAR DNA binding protein [TDP-43]) and mitochondrial proteins (superoxide dismutase 1 [SOD1], contributing to the neuromuscular dysfunction and motor neuron diseases [17,18]. Thus, improving mitochondrial function and suppression of oxidative stress are considered main strategies to prevent or intervene neurodegeneration related to aging or motor neuron diseases.

Among 3 types of protein arginine methyltransferases (Prmts), Prmt1 belongs to the type I Prmt and catalyzes asymmetric arginine dimethylation of histone and nonhistone substrates thereby modulating signaling pathways and gene expression related to diverse cellular events [15,19]. While Prmt1 knockout mice die early during embryogenesis [20], recent studies using mice lacking Prmt1 in muscle stem cells or myofibers have revealed essential roles of Prmt1 in muscle regeneration and maintenance [21]. However, the study on the role of Prmt1 in motor neurons is limited to in vitro models and still not well understood. Studies in motor neuron cell culture models have proposed a potential role of Prmt1 in ALS [22–25]. In primary motor neurons, Prmt1 was shown to be involved in ALS by interacting with FUS mutants to regulate their nucleo-cytoplasmic transport [24,26]. In the current study, we set out to determine the in vivo function of Prmt1 in motor neuron homeostasis and neuromuscular function using mice lacking Prmt1 in motor neurons mediated by Hb9-Cre (mnKO). The mnKO mice displayed age-associated motor neuron degeneration that led to muscle loss and midlife-onset lethality. The mice also exhibited elevated cellular stress response and mitochondrial dysfunction. The current study highlights the importance of Prmt1 in motor neurons and muscle maintenance, with implications for sarcopenia.

## Results

### Mice lacking Prmt1 in motor neurons exhibit age-related lethality and body weight loss

**Fig. 1.**
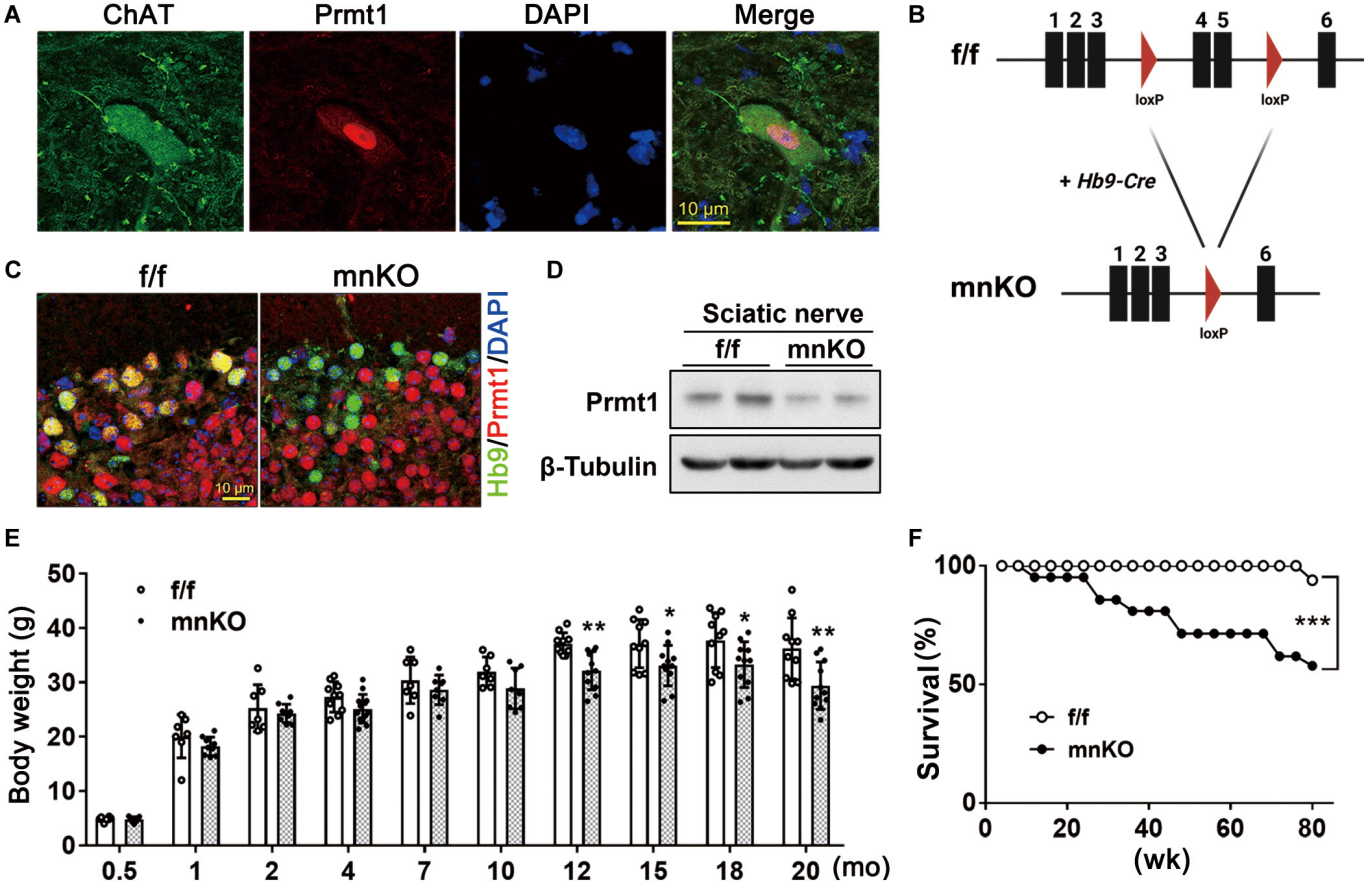
Prmt1 ablation in motor neurons causes lethality and body weight loss during aging. (A) Immunostaining of adult lumbar spinal cords for the expression of ChAT and Prmt1. (B) Schematic representation of the targeting strategy to generate motor neuron-specific Prmt1 deletion derived by Hb9-Cre mice. (C) Immunostaining of control Prmt1f/f (f/f) and Prmt1mnKO (mnKO) motor neurons at embryonic day 13.5 for Hb9 and Prmt1. (D) Immunoblot analysis of Prmt1 expression in sciatic nerve from f/f and mnKO mice. β-Tubulin serves as a loading control. (E) Body weight analysis of f/f and mnKO mice. Data are expressed as mean ± SD. (F) Survival curve of f/f (*n* = 33) and mnKO (*n* = 38) mice. To determine statistical significance, an unpaired 2-tailed Student *t* test was used. *P < 0.05; **P < 0.01; ***P < 0.001.

Prmt1 is expressed predominantly in the nuclei of choline acetyltransferase (ChAT)-positive motor neurons (Fig. 1A). To examine the role of Prmt1 in motor neurons, we generated motor neuron-specific Prmt1 knockout mice by crossing mice with 2 floxed-Prmt1 alleles (Prmt1^fl/fl^, f/f) with f/f mice hemizygous for Hb9-Cre expressing Cre recombinase under the control of a motor neuron-specific Hb9 promoter (Fig. 1B). The resulting f/f mice and f/f; Hb9-Cre^hemizygous^ (mnKO) littermates were used for phenotypic analysis. The ratio of f/f and mnKO mice was roughly equivalent and no overt developmental defects were observed (Fig. S1A). To confirm Prmt1 knockout in the motor neurons of mnKO, the neural tube sections of f/f and mnKO embryos at embryonic day 13.5 were subjected to immunostaining for Hb9 and Prmt1. Specific ablation of Prmt1 in Hb9-positive cells was observed in mnKO neural tubes while Prmt1 expression was maintained in other cell types such as glial cells (Fig. 1C and Fig. S1B). In addition, the sciatic nerves of 4-month-old mnKO mice showed reduced Prmt1 protein level compared to that of f/f mice (Fig. 1D). The residual Prmt1 expression of f/f mice are likely from other cell types such as Schwann cells in the sciatic nerves. The body weight of mnKO mice significantly declined starting around 12 months of age compared to the f/f littermates (Fig. 1E). Furthermore, mnKO mice exhibited increasing lethality with age and only about 50% survived up to 20 months of age (Fig. 1F). These results suggest that Prmt1 is dispensable for motor neuron development but important for motor neuron function in adult life.

### Young mnKO mice exhibit impaired motor function recovery after sciatic nerve injury

**Fig. 2.**
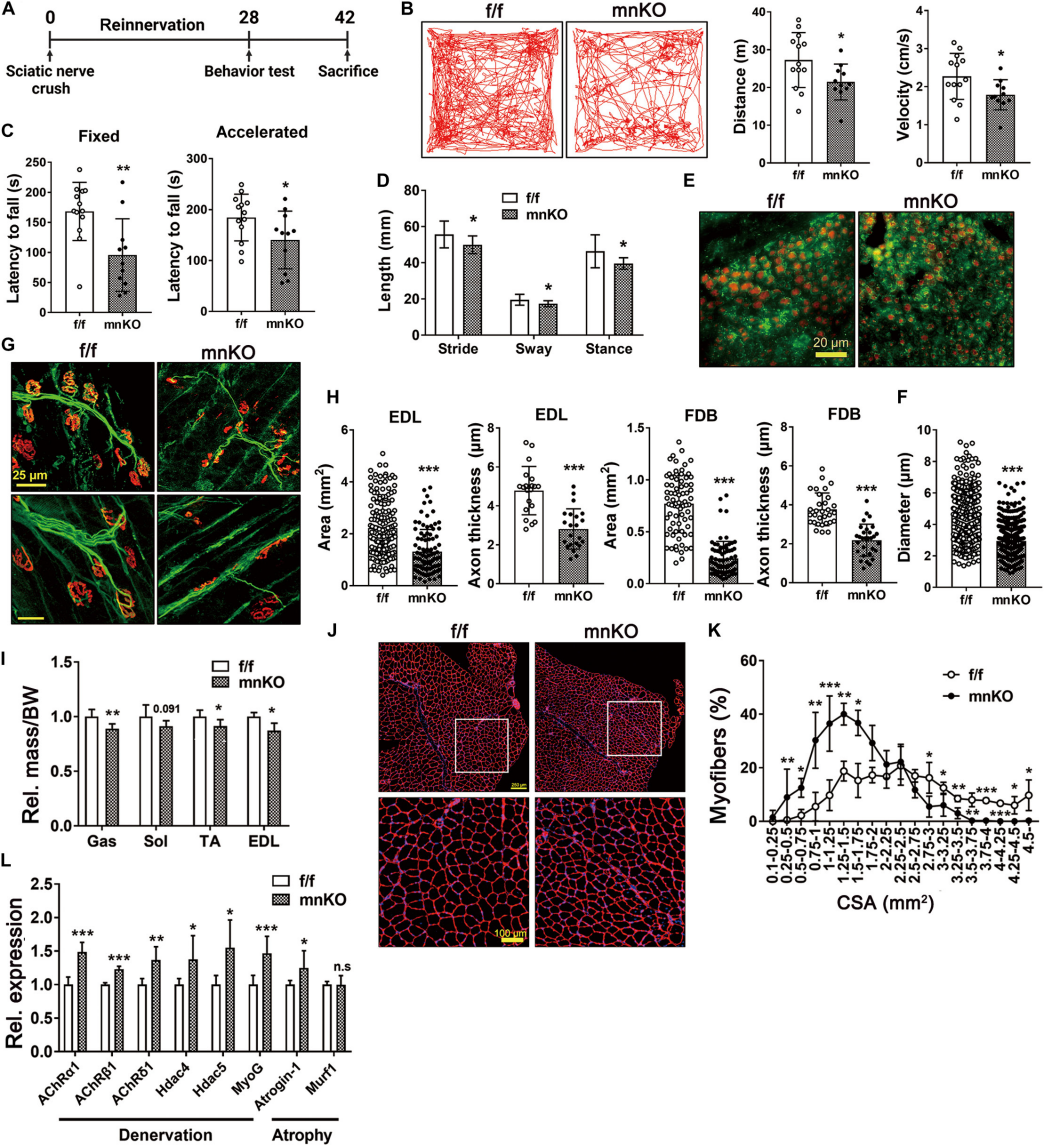
mnKO mice exhibit impaired motor function recovery post sciatic nerve injury. (A) Experimental scheme to assess neuromuscular recovery postsciatic nerve injury of 4-month-old mice. (B to D) Data from behavioral tests of f/f (*n* = 13) and mnKO (*n* = 11) mice at PID28: representative tracks of movement patterns and measured moved distance and velocity (B), fixed and accelerated rotarod test (C), and gait analysis (D). (E) Immunostaining for NF, red) and MBP, green) in f/f and mnKO sciatic nerves at 6 weeks of recovery. (F) Quantification of the axonal diameter. (G) Immunostaining for axon branch (NF, green), presynaptic (synaptophysin [SV], green) and postsynaptic (bungarotoxin [BTX], red) NMJ compartment in FDB and EDL muscles. (H) Quantification of the axon thickness and endplate area. Data are expressed as means ± SD. (I) The relative muscle mass to body weight of 4-month-old f/f (*n* = 4) and mnKO (*n* = 5) mice after injury. Data are expressed as means ± SD. (J and K) The assessment of myofiber CSA in the laminin-stained TA muscles. Quantification of myofiber CSA. Data are expressed as means ± SD. (L) qRT-PCR analysis for denervation and atrophy markers in denervated f/f (*n* = 3) and mnKO (*n* = 3) muscles. Values are means ± SEM. To determine statistical significance, an unpaired 2-tailed Student *t* test was used. *P < 0.05; **P < 0.01; ***P < 0.001.

Young mnKO mice did not exhibit noticeable deficits in motor function (Fig. S2A to D). Therefore, we set out to examine Prmt1’s role in nerve regeneration. 4-month-old f/f and mnKO mice were subjected to sciatic nerve crush injury, and motor function recovery of f/f and mnKO mice at post injury day 28 (PID28) was assessed by open field test, rotarod performance, and gait analysis (Fig. 2A). mnKO mice displayed significantly reduced locomotive activities evidenced by reduced distance moved and movement velocity compared to f/f mice (Fig. 2B). In addition, the rotarod performance with fixed or accelerated speed was substantially compromised in mnKO mice (Fig. 2C). Furthermore, mnKO mice exhibited reduced stride, sway, and stance length compared to f/f mice (Fig. 2D and Fig. S2E), implying compromised motor function recovery.

To assess the nerve regeneration at a tissue level following injury, sciatic nerves were harvested at PID42. Immunostaining for neurofilament (NF) and myelin binding protein (MBP) revealed a significantly smaller axon diameter of mnKO sciatic nerves than f/f sciatic nerves (Fig. 2E and F). Next, the morphology of NMJs in flexor digitorum brevis (FDB) and extensor digitorum longus (EDL) muscles at PID42 was examined. Muscles of f/f mice showed well-recovered NMJs with thick NF-positive axons and occupied endplates while mnKO muscles had mostly thin NF-positive axons and small unoccupied endplate of NMJs, implying impaired muscle reinnervation (Fig. 2G and H). Consistently, mnKO mice had a declined relative muscle mass to body weight compared to f/f mice (Fig. 2I). Also, tibialis anterior (TA) of mnKO exhibited a significant decrease in the cross-sectional area (CSA) of myofibers and elevated fibrosis by sirius red staining compared to TA muscle of f/f mice (Fig. 2J and K and Fig. S2F). Finally, the expression of denervation markers (acetylcholine receptor [AchR] α1, AchRβ1, AchRδ1, histone deacetylase [HDAC] 4, HDAC5, and myogenin) and a muscle-specific E3 ligase Atrogin-1 was significantly elevated in mnKO muscles compared to f/f muscles (Fig. 2L). Taken together, these data suggest that Prmt1 is required for the recovery of neuromuscular function after nerve injury.

### Old mnKO mice exhibit declined motor function and muscle mass

**Fig. 3.**
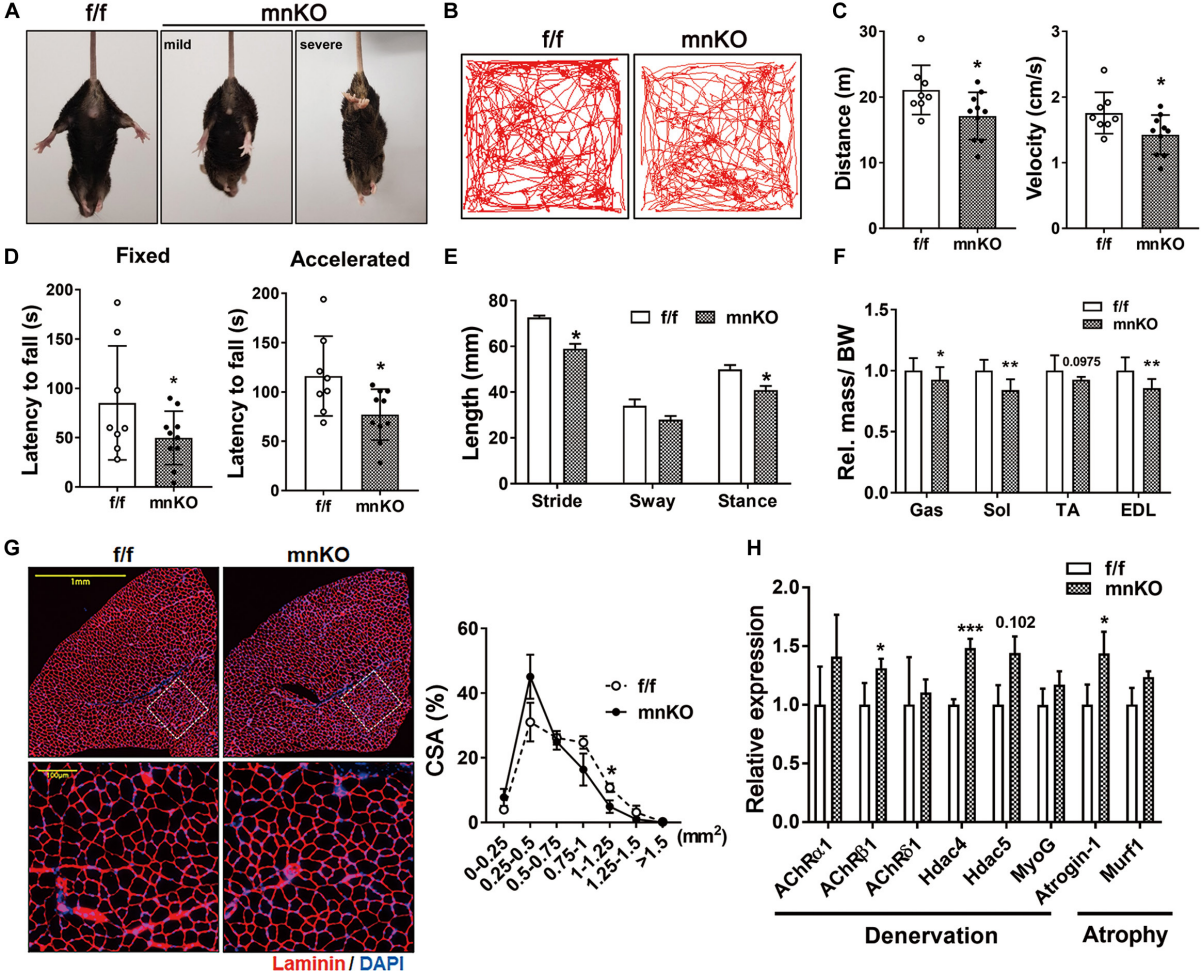
Old mnKO mice exhibit precociously declined motor function accompanied by nerve degeneration. (A) Analysis of hindlimb clasping in 20-month-old f/f and mnKO littermate mice. (B) The representative tracks of movement patterns of f/f and mnKO mice; the distance traveled is shown in red colors. (C) The physical activity tests showing distance moved and velocity. (D) The exercise activity on rotarod is assessed by fixed and accelerated rotarod speed. (E) Gait analysis of 20-month-old f/f (*n* = 2) and mnKO (*n* = 6) mice. (F) The relative muscle mass to body weight of 20-month-old f/f (*n* = 6) and mnKO (*n* = 6) mice. Data are expressed as means ± SD. (G) The assessment of myofiber CSA in the laminin stained TA muscles. Data are expressed as means ± SD. (H) qRT-PCR analysis for denervation and atrophy markers in aged f/f (*n* = 3) and mnKO (*n* = 3) muscles. Values are means ± SD. To determine statistical significance, an unpaired 2-tailed Student *t* test was used. *P < 0.05; **P < 0.01, ***P < 0.001.

To examine the underlying mechanisms of age-related lethality of mnKO mice, 20-month-old mnKO mice and their f/f littermates were subjected to the locomotion and motor coordination analyses. Most mnKO mice exhibited some degree of limb clasping indicative of muscle weakness, while f/f mice showed the normal splayed limb position (Fig. 3A). The open-field test revealed declined physical activities of mnKO mice measured by the distance moved and the movement velocity (Fig. 3B and C). To determine the forced motor activity of mice, the fixed or accelerated rotarod tests were utilized. mnKO mice showed substantially decreased latency to fall compared to f/f mice (Fig. 3D). Furthermore, mnKO mice exhibited alterations in gait behavior with significantly reduced stride and stance compared to f/f mice (Fig. 3E and Fig. S3A).

We then examined the weights of hindlimb muscles of 20-month-old mice. The relative muscle mass of mnKO mice was smaller than that of f/f mice (Fig. 3F). In a closer examination, the CSA of myofibers from mnKO TA muscles was substantially smaller compared to the f/f muscles with no dependency on the myofiber types (Fig. 3G and Fig. S3B to E). To determine the presynaptic Prmt1’s role on NMJ, the expression of the NMJ-related genes was compared between mnKO and f/f/ littermates. The expression of denervation markers (AchRβ1 and HDAC4) and an atrophy marker (Atrogin-1) was significantly altered in mnKO muscles compared to the f/f muscles (Fig. 3H). However, no significant changes were observed in mitochondrial DNA content and signaling pathways involved in protein synthesis other than the decrease in the phosphorylation of S6 kinase (Fig. S3F to H). These data imply Prmt1’s role in the motor neurons to maintain motor function and muscle mass during aging.

### Old mnKO mice exhibit abnormal NMJ morphology, axon degeneration, and decreased nerve conduction

**Fig. 4.**
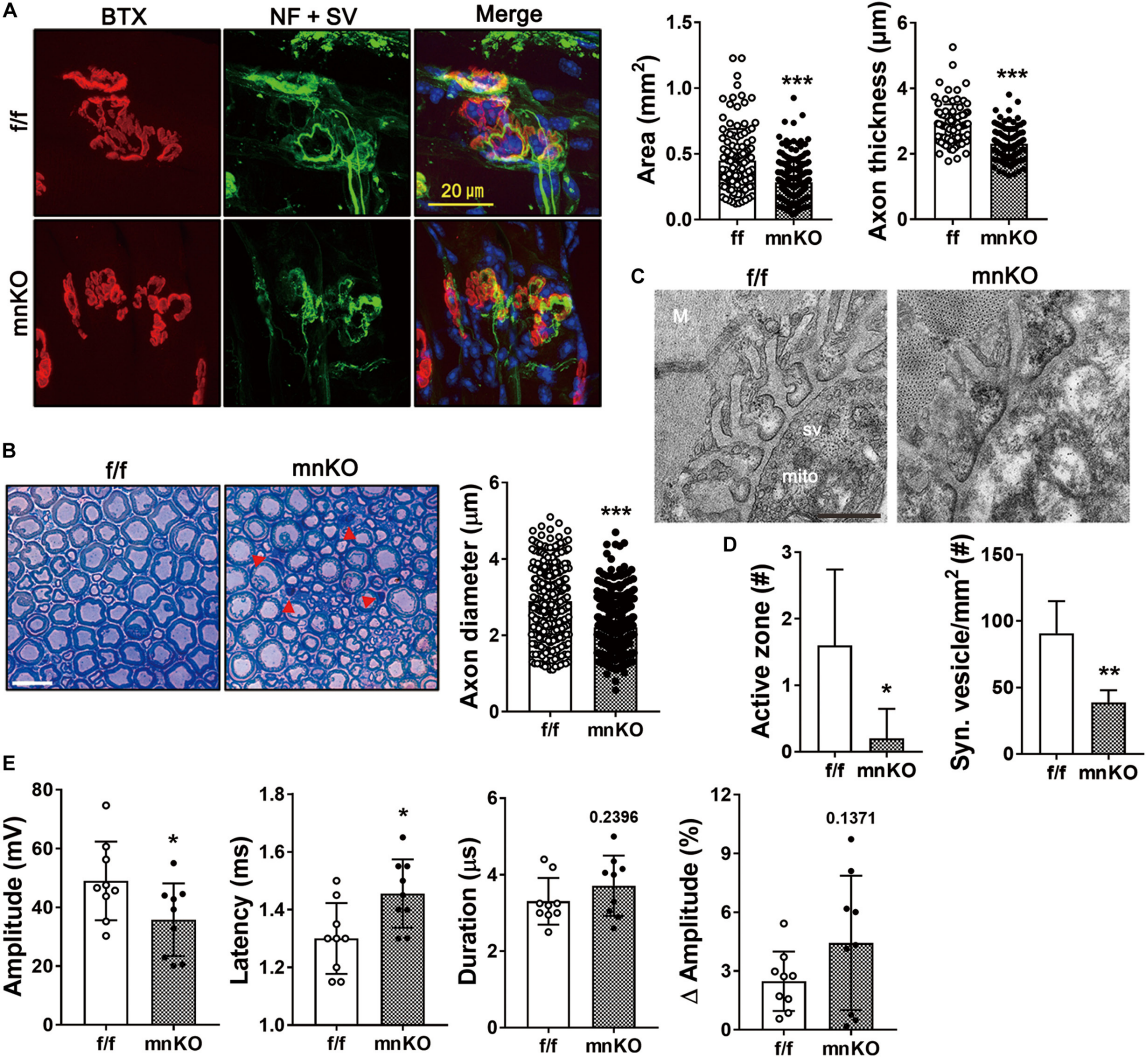
Aged mnKO mice exhibit muscle atrophy and NMJ abnormalities. (A) NMJ analysis by the immunostaining for presynaptic NF (green) and SV (green) and postsynaptic BTX (red) in FDB muscles. Quantification of the axon thickness and endplate area. Data are expressed as means ± SD. (B) Toluidine blue staining of sciatic nerve from aged f/f and mnKO mice. Quantification of average axon diameter per field. (C) Representative transmission electron microscopy images of NMJs in EDL muscles of aged f/f and mnKO mice. (D) Quantification of the synaptic active zone and vesicle number per field. Data are expressed as means ± SD. (E) CMAP analysis data showing average amplitude, latency, duration, and ∆ amplitude (ratio of the 10th to the first trace). Data are expressed as means ± SD. To determine statistical significance, an unpaired 2-tailed Student *t* test was used. *P < 0.05; **P < 0.01; ***P < 0.001.

To investigate how Prmt1 ablation in motor neurons leads to decline in motor function and muscle mass, NMJs and nerves of mnKO mice were examined. FDB from mnKO mice exhibited alterations in NMJ morphology with fragmentation, smaller size, and thinner axons. (Fig. 4A). The sciatic nerves of mnKO mice exhibited morphological alterations with degenerating axon fibers (marked with arrowhead) and significantly decreased average axon diameter compared to that of f/f mice (Fig. 4B). In transmission electron microscopy, NMJs in f/f muscles showed a well-organized synaptic area with accumulated synaptic vesicles and active zones, while mnKO NMJs exhibited abnormal morphology with declined presynaptic vesicles and abnormal mitochondria (Fig. 4C and D). To determine the nerve conduction, we assessed compound muscle action potentials (CMAPs) from TA muscles. The amplitude of CMAPs was significantly lower in mnKO mice, while the latency was significantly higher without significant alterations in duration and amplitude compared to f/f mice (Fig. 4E). These data together suggest that Prmt1 deficiency causes alterations in nerve–muscle conduction accompanied by changes in NMJ structure and axon degeneration.

### Prmt1 ablation in motor neurons causes alteration in transcriptome of genes related to inflammation, apoptosis, and mitochondrial dysfunction

**Fig. 5.**
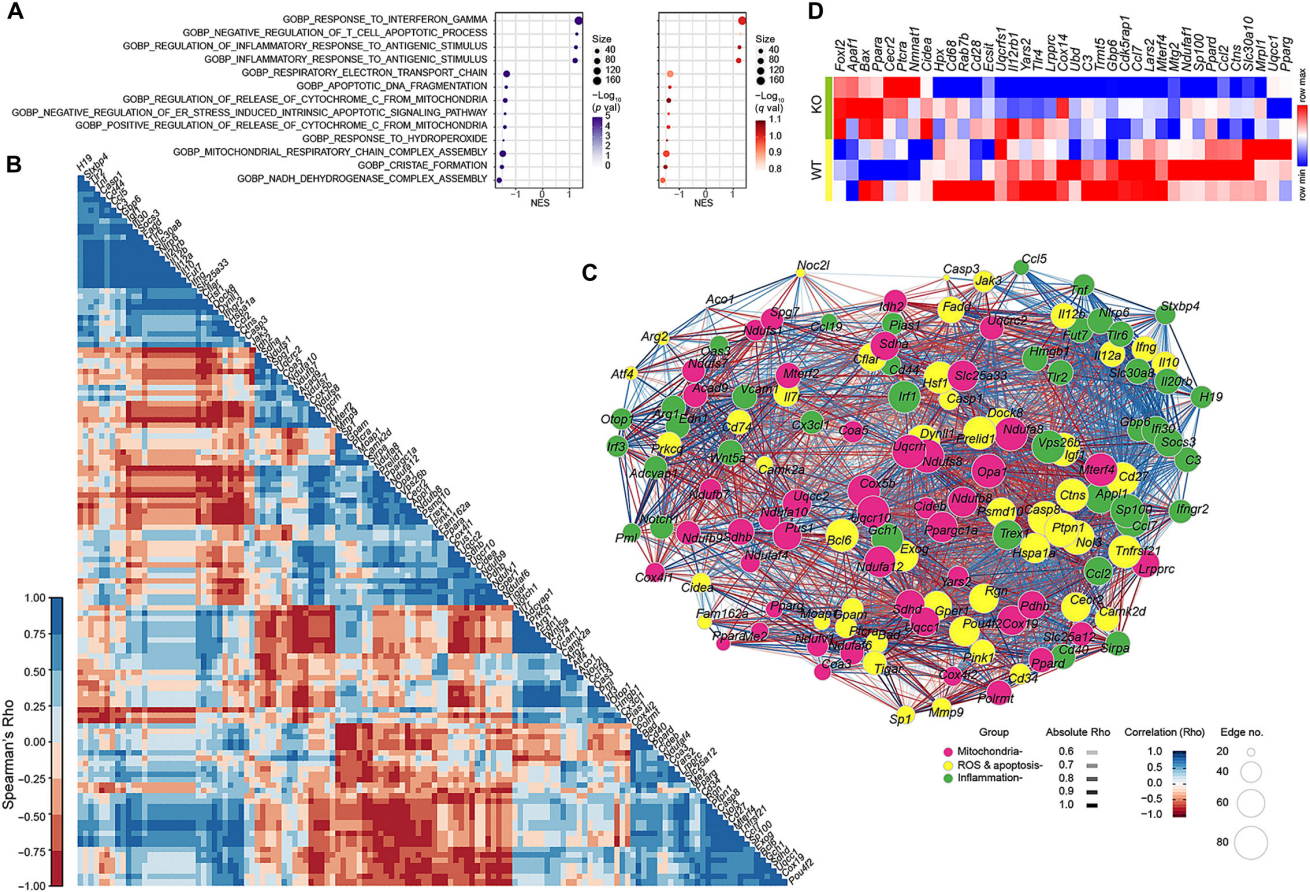
Alteration of gene expression profiles in mnKO mouse spinal cords. (A) The bubble plots present the nominal *P* values (purple) and false discovery rates (FDR, *q* values) of the top correlated gene set. The X-axis indicates normalized enrichment scores (NES) of GSEA. The depth of the shading in each bubble indicates the magnitude of *P* or *q* values. (B) Correlogram matrices display Spearman’s Rho of 2 genes facing each side of the square (matrix). The shading intensity of the correlation matrices displays Spearman's Rho displayed in the scale (left-hand side of the correlogram). (C) Gene network shows coexpression of genes involved in top correlated gene sets (presented in Fig. 5A). The Spearman's Rho between 2 nodes (genes) generates the color and depth of each edge. The color (pink, yellow, and green) of each node indicates mitochondria-, ROS and apoptosis-, or inflammation-related gene sets, respectively. The size of a node indicates the number of connected (correlated) nodes (Spearman’s Rho > 0.5). (D) Heatmap shows the expression of representative genes in those gene sets presented in the (A).

To reveal major pathways involved in the age-related motor neuron defects in mnKO mice, an unbiased gene set enrichment analysis (GSEA) was performed using the transcriptomes of lumbar spinal cords from 22-month-old f/f and mnKO mice. Interestingly, the gene sets of inflammation, mitochondria, reactive oxygen species (ROS), and apoptosis were ranked at the top of the list with a statistical significance (nominal *P* value < 0.05 and false discovery rate *q* value < 0.25) (Fig. 5A). Spearman's rank correlation analysis and gene network analysis showed that the genes of the listed gene sets were also tightly correlated with one another, implying that the altered expressions of those genes were not generated randomly in mnKO mice (Fig. 5B and C). In particular, heatmap indicated altered expression of the representative genes associated with mitochondria and oxidative stress response in mnKO spinal cords (Fig. 5D), suggesting that the cellular stress caused by mitochondrial dysfunction may be the major mechanism contributing to neurodegeneration in mnKO mice.

### Prmt1 ablation augments cellular stress responses in motor neurons

**Fig. 6.**
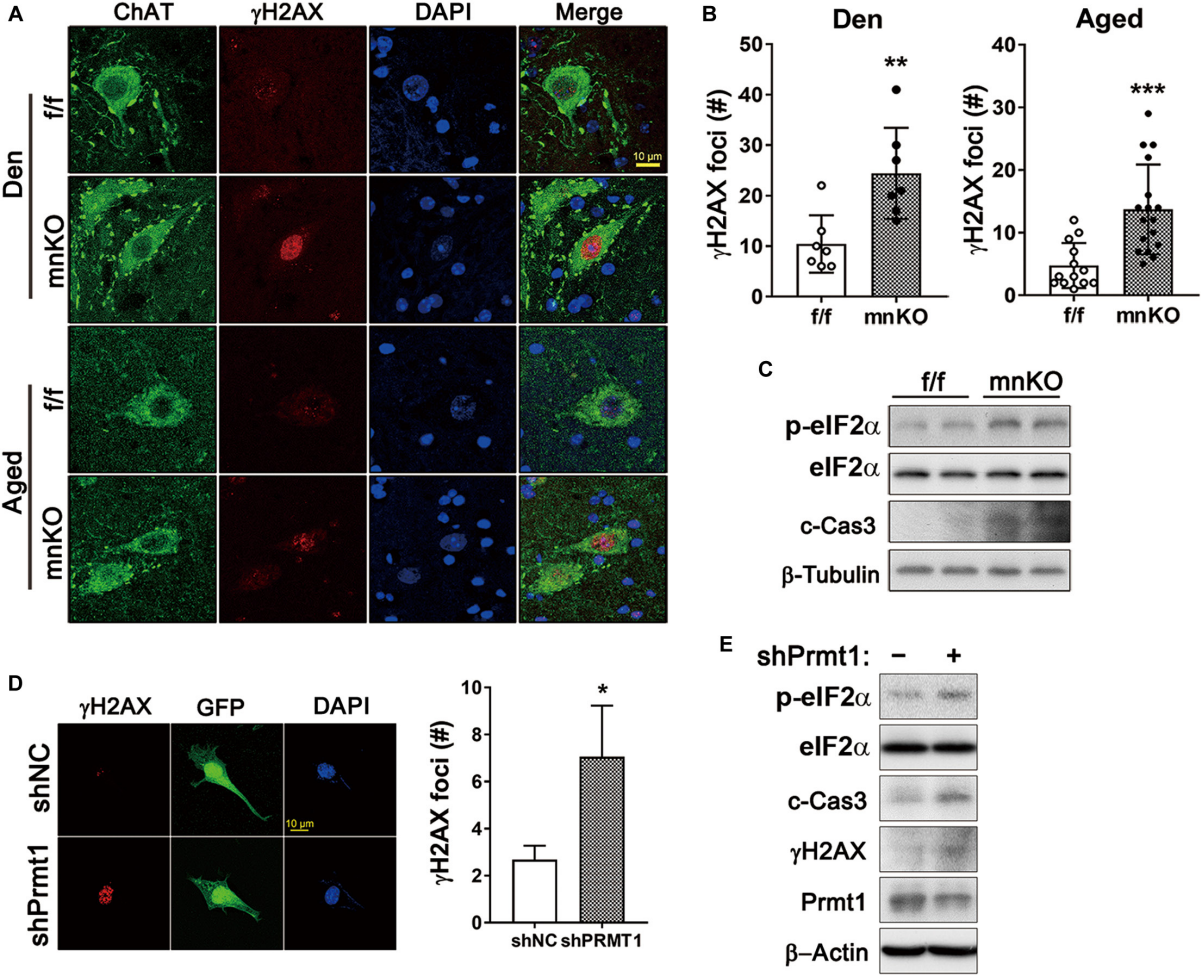
Prmt1 depletion augments cellular stress in motor neurons. (A) Immunostaining for ChAT and γH2AX in motor neurons from denervated/regenerating (Den) and aged f/f and mnKO mice. (B) Quantification of γH2AX foci per motor neuron. Data are expressed as means ± SD. (C) Immunoblot analysis for p-eIF2α, eIF2α, c-Cas3, and β-actin in aged lumbar spinal cords of f/f and mnKO mice. (D) Immunostaining for γH2AX in NSC34 cells expressing shNC or shPrmt1 marked by GFP expression. Quantification of γH2AX foci number per field. Data are expressed as means ± SD. (E) Immunoblot analysis for p-eIF2α, eIF2α, cleaved-Caspase 3 (c-Cas3), γH2AX, Prmt1, and β-actin in NSC34/shNC and NSC34/shPrmt1 cells. To determine statistical significance, an unpaired 2-tailed Student *t* test was used. *P < 0.05; **P < 0.01; ***P < 0.001.

To examine the cellular stress in mnKO motor neurons, lumbar spinal cords of mnKO mice at PID42 (denervation) and 20-month-old mnKO mice (aged) were subjected to the immunostaining for ChAT and γH2AX (the phosphorylated form of H2AX). ChAT-positive motor neurons from the aged or the injury-recovering mnKO lumbar spinal cords had significantly elevated γH2AX-positive foci per cell compared to f/f motor neurons (Fig. 6A and B). In addition, the lumbar spinal cords of aged mnKO mice showed increased protein levels of phosphorylated eukaryotic initiation factor 2α (p-eIF2α) and cleaved-Caspase 3 (c-Cas3) compared to that of f/f mice (Fig. 6C). To verify, Prmt1 depletion in NSC34 motor neuron-like cells was carried out, followed by γH2AX immunostaining and immunoblotting for stress-related proteins. Consistent with the in vivo results, Prmt1 depletion in NSC34 cells increased the number of γH2AX-positive foci (Fig. 6D). In addition, the levels of p-eIF2α, c-Cas3, and γH2AX were elevated in Prmt1-depleted NSC34 cells (Fig. 6E). These results imply a protective role of Prmt1 in motor neurons against cellular stress.

### Prmt1 inhibition decreases mitochondrial activity in motor neurons

**Fig. 7.**
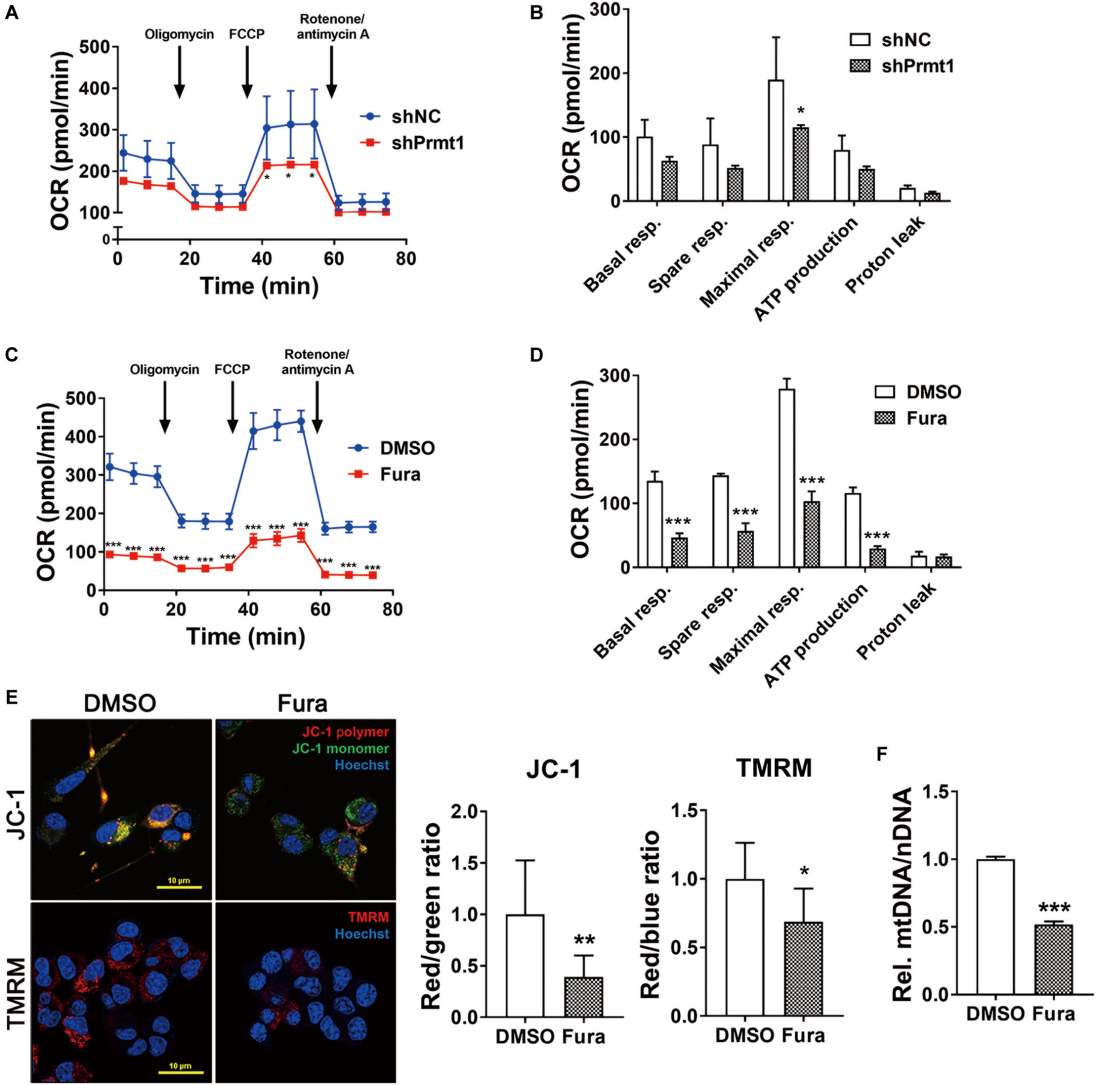
Prmt1 inhibition causes mitochondrial dysfunction in motor neurons. (A) Oxygen consumption rate (OCR) for mitochondrial respiration activity in NSC34 cells expressing shNC or shPrmt1. (B) Quantification of OCR. Data are expressed as means ± SD. (C) Oxygen consumption rate (OCR) for mitochondrial respiration activity in DMSO or Furamidine-treated NSC34 motor neuron cells. (D) Quantification of OCR. Data are expressed as means ± SD. (E) JC-1 and TMRM staining of mitochondria membrane potential in DMSO or Furamidine-treated NSC34 motor neuron cells. Quantification of JC-1 polymer/monomer ratio per cell and TMRM signaling per cell. Data are expressed as means ± SD. (F) The relative mitochondrial DNA (mtDNA)–to–nuclear DNA (nDNA) ratio. The values from control were set to 1. Values are means ± SD. To determine statistical significance, an unpaired 2-tailed Student *t* test was used. *P < 0.05, **P < 0.01, ***P < 0.001.

To examine the effect of Prmt1 inhibition on mitochondrial function of motor neurons, NSC34 cells were treated with Prmt1-specific inhibitor, Furamidine (Fura) for 24 h or transduced with adenoviral Prmt1shRNA followed by mitochondrial activity analysis using seahorse analyzer. Prmt1 inhibition by Fura treatment or Prmt1shRNA transduction in NSC34 cells resulted in lower basal respiration rate than controls (Fig. 7A to D). In addition, the spare and FCCP (carbonylcyanide-p-trifluoromethoxyphenylhydrazone)–induced maximal respiration and adenosine triphosphate production were decreased in Prmt1-inhibited cells relative to the control cells. To determine whether the decrease in mitochondrial activity is simply due to reduced mitochondrial content or contributed by mitochondrial dysfunction, NSC34 cells treated with DMSO or Fura for 24 h were subjected to analysis for the mitochondrial membrane potential using the fluorescent dye JC-1 or TMRM (tetramethylrhodamine methyl ester) (Fig. 7E). Fura-treated cells displayed decline in the red/green ratio for JC-1 staining and TMRM signals, indicative of decreased mitochondrial function. Also, Fura-treated NSC34 cells exhibited significantly declined mitochondrial DNA content, compared to the control cells (Fig. 7F). The decrease in mitochondrial content following Prmt1 inhibition was confirmed in vivo by Tomm20 staining and Western blot analysis of mnKO lumbar spinal cord (Fig. S4A and B). These data imply that Prmt1 depletion in motor neurons results in decreased mitochondrial function, leading to increase in cellular oxidative stress.

### Prmt1 overexpression alleviates TNFα-induced stress response in motor neurons

**Fig. 8.**
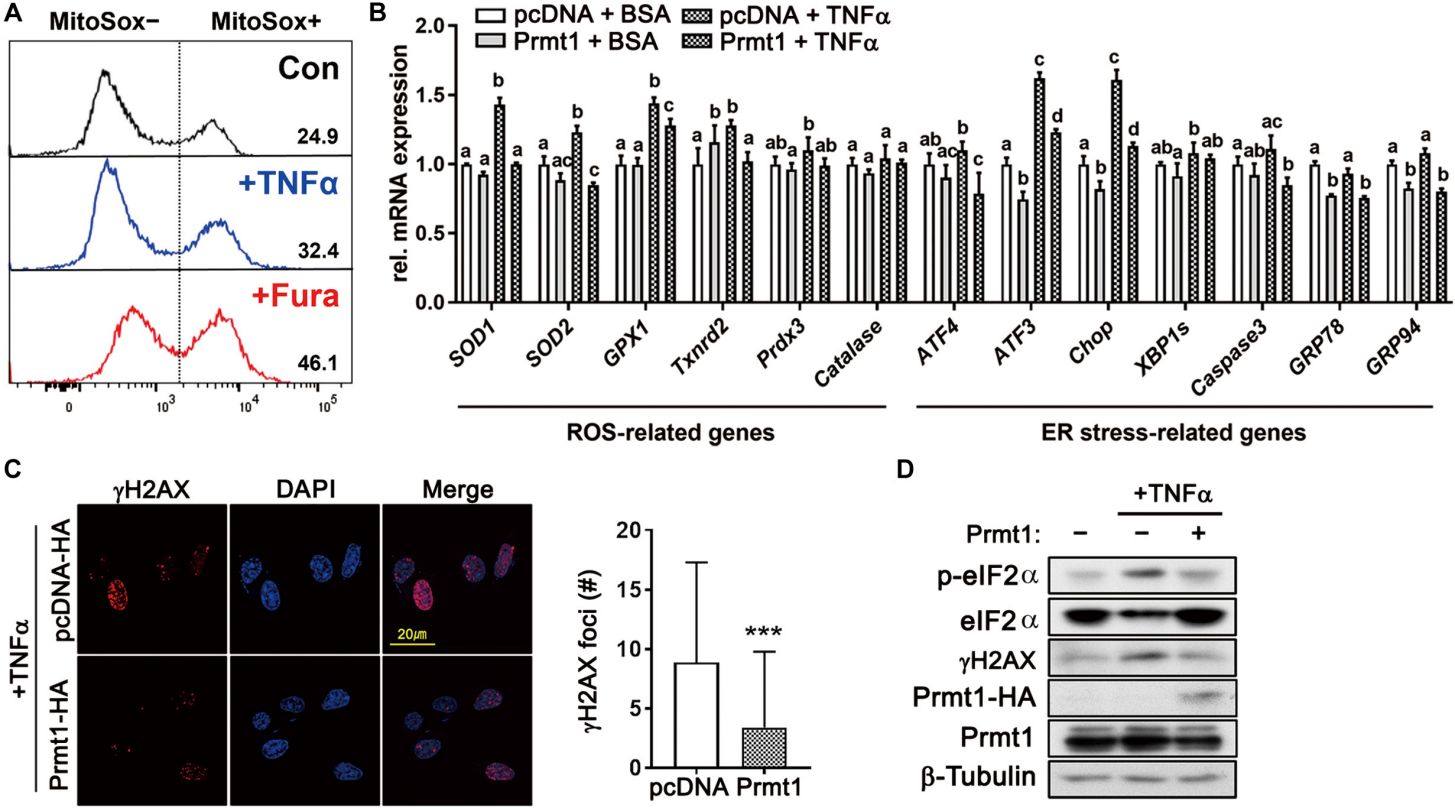
Prmt1 overexpression attenuates cellular stress in response to TNFα. (A) Flow cytometric analysis of MitoSox-positive cells in NSC34 motor neuron cells treated with DMSO/BSA (Con), DMSO/TNFα (TNFα), and Furamidine/BSA (Fura). (B) qRT-PCR analysis of ROS- and ER-stress-related genes of NSC34 motor neurons in response to TNFα (50 ng/ml) treatment for 6 h. Data are expressed as means ± SD. To determine statistical significance, a 2-way ANOVA was used. Same alphabetical notation above the bars indicates no statistical significance, while different alphabetical notation indicates statistical significance with *P* value lower than 0.05. (C) Immunostaining for γH2AX and DAPI in NSC34/pcDNA-HA and NSC34/Prmt1-HA cells treated with TNFα. Quantification of γH2AX foci number per nucleus. Data are expressed as means ± SD. To determine statistical significance, an unpaired 2-tailed Student *t* test was used. (D) Immunoblot analysis for p-eIF2α, eIF2α, γH2AX, Prmt1-HA, and β-tubulin in NSC34/pcDNA-HA and NSC34/Prmt1-HA cells treated with TNFα. *P <0.05, **P <0.01, ***P < 0.001.

Since our data imply a protective role of Prmt1 in motor neurons against cellular stress response, we analyzed the effect of Prmt1 overexpression in motor neurons on TNFα (tumor necrosis factor α)–triggered oxidative stress. Flow cytometric analysis of MitoSox-positive cells revealed increased oxidative stress in NSC34 motor neurons in response to either TNFα or Fura treatment (Fig. 8A). Also, TNFα treatment induced elevated levels of both oxidative stress- or ER stress-related gene expressions in NSC34 cells (Fig. 8B). However, Prmt1 overexpression attenuated this increase in the expression of stress-related genes in response to TNFα. Prmt1 overexpression also ameliorated the increase in the number of γH2AX-positive foci and the level of p-eIF2α following TNFα treatment (Fig. 8C and D). These data further support the role of Prmt1 in protecting motor neurons against cellular stress. Taken all together, our study demonstrates that Prmt1 is a critical regulator for the maintenance of motor neuron function via modulation of mitochondrial function.

## Discussion

Prmt1 in motor neurons have been mostly studied in primary culture of motor neurons or motor neuron-like cell lines in vitro. To our knowledge, Prmt1 mnKO mice offer the first in vivo evidence of PRMT1’s role in age-related degeneration of motor neurons. mnKO mice exhibited midlife-onset lethality, motor neuron degeneration, and a decline in muscle mass and function with age. Additionally, motor neuron-specific Prmt1 ablation impaired motor function recovery post sciatic nerve crush-triggered denervation in young animals. These defects were accompanied by structural abnormalities of NMJs, which are a common feature associated with aging-related neuromuscular degeneration [6,9,27], suggesting that Prmt1 is a critical regulator for the maintenance of motor neurons during aging.

Prmt1 has been shown to be sequestered to the cytosolic ribonucleic protein aggregates formed by ALS-linked FUS mutants in motor neurons [24]. This has been suggested as one of the potential mechanisms of neurotoxicity caused by ALS-FUS mutants as they prevent PRMT1’s endogenous function in the nucleus. However, ALS-FUS transgenic mice displays severe early onset motor impairment accompanied by the loss of spinal motor neurons [28], while mnKO mice from our study displayed a much slower and progressive disease phenotype with age. This suggests that the cytosolic sequestration of PRMT1 can only partially explain the early onset pathology of ALS-FUS transgenic mice and the ALS-FUS induced neurotoxicity is multilayered.

Oxidative stress, inflammation, and mitochondrial dysfunction have been shown to be critical factors in aging-related motor neuron degeneration and neuromuscular diseases (NMDs) [6,7,11,16,19,29,30]. The expression of genes related to oxidative stress, inflammation, and mitochondria was greatly altered in mnKO spinal cords compared to that in WT spinal cords. In addition, Prmt1 deficiency caused elevated γH2AX-positive motor neurons in lumbar spinal cords of aged mice or nerve-crush models, as well as in the Prmt1 knockdown or Furamidine treated NSC34 motor neurons. TNFα-induced cellular stress response was attenuated by Prmt1 overexpression in motor neurons, further suggesting a protective role of Prmt1 against cellular stress. Thus, protein arginine methylation mediated by Prmt1 plays a critical, nonredundant role in the protection against cellular stress in motor neurons.

Mitochondrial function declines with age, and dysfunctional mitochondria produce increased level of ROS [7]. The oxidative stress induced by ROS, in turn, damages mitochondria, forming a vicious cycle that is one of the major factors in the etiology of age-associated diseases such as sarcopenia [7,19,31]. Recent studies have suggested a role of Prmt1 in mitochondrial energy metabolism/homeostasis in multiple cellular systems [32,33]. Depletion of Prmt1 in C. elegans causes mitochondria dysfunction with decreased adenosine triphosphate and increased ROS production [32]. In lung fibroblasts and hepatic cells, Prmt1 is implicated in maintaining homeostasis of mitochondrial mass [33]. In HeLa cells, Prmt1 modulates mitochondrial translocation via methylation of apurinic/apyrimidinic endonuclease 1 to protect against oxidative damage [20]. In primary neurons, Prmt1 inhibition leads to fragmented mitochondria [24]. In line with this, mnKO spinal cord showed signs of decrease in mitochondrial mass and activity, which were also supported by in vitro analysis. Our study further underlines the importance of Prmt1 in mitochondrial homeostasis and suggests that elevated cellular stress caused by mitochondrial dysfunction may be a potential mechanism of pathological phenotype observed in mnKO mice.

Studies on Prmt1 in relation to NMDs have mostly focused on its interaction with RNA-binding proteins with ALS-associated mutations [17,22]. This study spotlights Prmt1’s role in the maintenance of neuromuscular function during aging independent of the ALS-associated mutations. Previously, we showed that skeletal muscle specific KO of Prmt1 leads to muscle atrophy from 6-months of age due to upregulation of autophagy pathway [19]. Prmt1’s functional significance in both motor neurons and skeletal muscle further signifies the enzyme’s relevance in the etiology of NMDs and its potential as a therapeutic target. Future studies on the underlying mechanism of Prmt1’s involvement in NMDs will offer exciting opportunities in developing treatments for NMDs.

## Materials and Methods

### Animal studies

Mice bearing the Prmt1-floxed allele (Prmt1^fl/fl^) were maintained as previously described [19]. To generate mice with a motor neuron specific ablation of Prmt1, Prmt1^fl/fl^ (f/f) were crossed with transgenic mice expressing Cre under the control of the Hb9 promoter (Hb9-Cre, Jackson Laboratory). Mice lacking Prmt1 in motor neurons (mnKO) were generated by crossing Prmt1f/f with Prmt1^fl/fl^ containing a single copy of Hb9-Cre. To examine the phenotypes and other molecular mechanisms, f/f and mnKO littermates were used.

For the sciatic nerve crush injury, mice were anesthetized with isoflurane and the skin was incised posterior and parallel to the left femur. The sciatic nerve was exposed at mid-thigh and crushed proximal to its branching using a forceps for 10 s. Following the crush injury, the nerve was repositioned, and the muscle and skin incision were closed with a surgical dissolvable suture and a suture tape, respectively. Carprofen (5 mg/kg) was administered subcutaneously for the postoperative pain control. All animal experiments were reviewed and approved by the Institutional Animal Care and Use Committee at Sungkyunkwan University (SKKU) and complied with the animal experiment guidelines of SKKU Ethics Committee (SKKUIACUC2021-06-14-1). All experimental mice were housed in the animal facility at 23 °C with a 12:12-h light–dark cycle with ad libitum access to food and water.

### Open-field, rotarod, footprint test

To analyze the behavioral difference between f/f and mnKO mice, the open-field, foot print, and rotarod tests were performed sequentially with 2 to 3 d of interval between the tests in a blinded fashion. All behavioral tasks were performed during the light phase. All objects used in behavioral analysis were cleaned with ethanol between trials. The open-field test was carried out in an open plastic container (30 × 30 × 30 cm). At the start of each trial, mice were placed in a corner of the plastic container, and their movement was recorded for 20 min. The Noldus EthoVision 13.0 tracking software was used to analyze the moved distance and velocity. A rotarod apparatus (model 7600; Ugo Basile) was used to measure motor coordination and balance. For the fixed speed rotarod test (13 rpm) and the accelerating rotarod test (5 to 18 rpm over 40 s), mice were given 3 practice trials for 3 d before the real experiment. A 200-s cutoff per session was used. The latency to fall off the rotarod was recorded for both configurations. Mice that rotated passively were scored as fallen.

To obtain footprints, the hindfeet of mice were coated with black nontoxic paint. The animals were then allowed to walk along a 50-cm-long, 10-cm-wide runway (with 10-cm-high walls) into an enclosed box, and a sheet of white paper was placed on the floor of the runway. To characterize the walking pattern of each mouse, the average distance between each stride (stride length), left and right hind footprints (sway length), and left and right front footprints (stance length) was measured.

### Cell culture experiments

NSC34 motor neuron-like cells were cultured in growth medium (DMEM high glucose, Gibco) containing 10% fetal bovine serum (Gibco), 1% glutamax (Gibco), 10 units/ml penicillin, and 10 μg/ml streptomycin (Welgene Inc.) at 37 °C, 5% CO_2_. Cells were transfected using TransIT-2020 Transfection Reagent (Mirus, MIR5406). To deplete or overexpress Prmt1, the expression vectors for Prmt1 or Prmt1 shRNA with matched controls were utilized as previously described [34].

### Protein experiments

Immunoblot experiments were performed as previously described [35]. Briefly, cells or homogenized tissue samples were dissolved in RIPA buffer (1× phosphate-buffered saline, 1% IGEPAL CA-630 [v/v], 0.1% sodium dodecyl sulfate [SDS] [w/v], 0.5% sodium deoxycholate [w/v], and 50 mM sodium fluoride). Primary antibodies for immunoblot used in this study are provided in Table S2.

### Histology and immunostaining

Spinal cord and hindlimb muscle tissues were embedded in Tissue-Tek OCT Compound (Sakura), and 7-μm-thick serial sections were prepared using a cryomicrotome. To assess the structure and fibrosis, fixed cryosections were stained with Picro-Sirius red (Abcam) according to the manufacturer’s protocol. To examine the axon recovery post sciatic nerve crush injury, sciatic nerve sections were immunostained with NF (NF; 837904, BioLegend) and myelin binding (MBP; ab40390, Abcam) antibodies. Spinal cords were immunostained with Prmt1 (07-404, Millipore), Hb9 (PA5-23407, Invitrogen), Tomm20 (H00009804-M01, Abnova), gamma H2AX (Ser139) (NB100-384, Novus), GFAP (13-0300, Invitrogen), and ChAT (AB144P, Chemicon). Muscle histology was examined as previously described [36,37]. To assess myofiber size, muscle sections were immunostained with primary antibodies against laminin (ab11575, Abcam). Fluorescent images were obtained by using the LSM-710 confocal microscope system (Carl Zeiss) and ZEN software (Carl Zeiss) and analyzed with ImageJ software

For NMJ immunostaining, whole muscle and single myofiber were fixed, permeabilized, and incubated with primary antibodies against NF-H (801601, BioLegend), NF (837904, BioLegend), synaptophysin (SV, Developmental Studies Hybridoma Bank), and tetramethylrhodamine alpha-bungarotoxin (BTX; B35451, Invitrogen) and secondary antibodies (Life Technologies, A-11001 and A-11012). The images were captured and processed with a Nikon ECLIPSE TE-2000U inverted microscope using NIS-Elements F software (Nikon), and the myofiber area was measured using ImageJ software. For muscle histology, the cryosections were stained with Mayer's hematoxylin and eosin (BBC Biomedical). The images were captured using a Nikon ECLIPS TE-2000U inverted microscope and Tissue FAXS imaging software (TissueGnostics).

### Electron microscopy and toluidine staining

For sciatic nerve isolation, the mice were perfused with 4% paraformaldehyde before sacrifice. A segment of the peripheral nerve was excised 1 mm distal to the point of separation from the main branch point of the sciatic nerve and drop-fixed in modified Karnovsky's fixative (20% paraformaldehyde and 8% glutaraldehyde in 0.2 M sodium cacodylate, pH 7.4) at 4 °C overnight. The tissue was washed with 0.2 M sodium cacodylate buffer (pH 7.2) and post-fixed with 2% aqueous osmium tetroxide for 2 h. The samples were then washed with cacodylate buffer, dehydrated through ascending alcohols, washed with propylene oxide, and embedded in Spurr’s resin (Electron Microscopy Sciences). For light microscopy, 500-nm semithin sections were prepared on an EM U7 microtome (Leica), counterstained with toluidine blue, and viewed on a light microscope AxioImager M2 (Carl Zeiss). Images were recorded with an AxioCam MRm. For transmission electron microscopy (TEM), ultrathin sections (70 to 80 nm) were prepared on an EM UC7 microtome (Leica) and collected on 1 × 2 mm formvar coated copper slot grids. Images were captured with a Talos L120C (FEI). Data were analyzed using Image J software.

### CMAPs

Measurement of CMAPs was performed as previously described [38] with minor modification. Mice were anesthesized with 5% isoflurane and maintained by continuous inhalation of 2% isoflurane mixed with oxygen. For sciatic nerve stimulation and tracing CMAPs, a portable electromyography unit (PowerLab, AD instruments) was used. The active electrodes were positioned at TA muscle. Sciatic nerve stimulation was performed using a voltage of 5, 10, and 20 mV and repeated 10 times each. Four parameters (Amplitude, Duration, Latency, and amplitude change) after each stimulation was recorded by the LabChart software version 8.0 (AD instruments).

### RNA analysis

Quantitative reverse transcription polymerase chain reaction (qRT-PCR) analysis was performed as previously described [35,39]. Briefly, the tissues were homogenized by FastPrepR-24 (MP Biomedicals) and extracted using an easy-spin Total RNA Extraction Kit (iNtRON Biotechnology). The fold change in gene expression was normalized against the expression of large ribosomal subunit β-actin (*Actb*). The sequences of the primers used in this study are provided in Table S1.

To profile the global alteration of transcriptome in mnKO spinal cords, RNA sequencing was performed with total RNA prepared from the spinal cords of postnatal day1 f/f and mnKO mice with Agilent 2100 Bioanalyzer using the RNA 6000 Nano Chip (Agilent Technologies). RNA sequencing data were analyzed by using ExDEGAv1.61 (e-Biogen, Korea). The global and functional alterations of gene expression were evaluated with the unbiased GSEA (Broad Institute; www.broadinstitute.org/gsea) with the GSEA software (v4.1.0 Java Web Start) and visualized as described previously [35,40]. All plots were generated with RStudio (RStudio Desktop 1.4.1106 for Windows) containing R (R-4.0.5 for Windows) and R packages including ggpubr, ggplot2, igraph, ggraph, egg, corrr, corrplot, dplyr, tidyverse, and reshape (www.r-project.org; www.rstudio.com/).

### Mitochondrial analysis

To analyze mitochondrial respiration, NSC34 cells were seeded on collagen-coated coverglass bottom dishes at a density of 6 × 104 cells/ml, allowed to grow for a day, and treated with furamidine (20 μM; a Prmt1-specific inhibitor) for 24 h. The cells were then stained with 2.0 μg/ml JC-1 Dyes (MP 03168, Invitrogen) for 30 min, washed with Live Cell Imaging Solution (A14291DJ, Invitrogen), and imaged via a confocal microscope system (Carl Zeiss). For mitochondrial DNA contents, total DNA was extracted from DMSO- or furamidine-treated NSC34 cells using DNeasy Blood and Tissue kit (QIAGEN). The amount of mitochondrial DNA was quantified by the ratio of Mtco1 to β-actin by quantitative PCR. Mitochondrial respiratory activity was measured in live cells by using Seahorse XF HS Mini (Agilent Technologies) according to the manufacturer's protocol.

### Statistics

Values are expressed as means ± SD or ± SEM, as indicated in the figure legends. The statistical significance was calculated using either Student *t* test (unpaired, 2-tailed) or analysis of variance (ANOVA) followed by post hoc Tukey's tests for multiple comparisons. Differences were considered statistically significant if *P* < 0.05. All graphs and statistical analyses were performed using GraphPad Prism software version 8.0.

## Data Availability

The authors declare that the data supporting the findings of this study are available within the paper and its supporting information files or on request from the corresponding author.

## Supplementary Materials

Fig. S1. Motor neuron-specific ablation of Prmt1. Fig. S2. mnKO mice exhibit impaired motor function recovery and muscle degradation after post sciatic nerve injury. Fig. S3. Aged mnKO mice shows impaired muscle function. Fig. S4. Mitochondrial content is decreased in motor neurons of aged mnKO mice. Table S1. Sequences of qRT-PCR primers. Table S2. Information of immunoblot analysis.
