# Supplementary material for: Protein Arginine Methyltransferase 1 Ablation in Motor Neurons Causes Mitochondrial Dysfunction Leading to Age-related Motor Neuron Degeneration with Muscle Loss
Source: Research (Wash D C). 2023 Jun 19;6:0158. doi: 10.34133/research.0158 (PMC10278992; doi:10.34133/research.0158)
Supplement: Supplementary file 1 — Fig. S1. Motor neuron-specific ablation of Prmt1. Fig. S2. mnKO mice exhibit impaired motor function recovery and muscle degradation after post sciatic nerve injury. Fig. S3. Aged mnKO mice shows impaired muscle function. Fig. S4. Mitochondrial content is decreased in motor neurons of aged mnKO mice. Table S1. Sequences of qRT-PCR primers. Table S2. Information of immunoblot analysis. [file research.0158.f1.docx]

**Front Matter**

Title

Protein arginine methyltransferase 1 ablation in motor neurons causes mitochondrial dysfunction leading to age-related motor neuron degeneration with muscle loss

**Authors**

Hyun-Kyung So^1,2^†, Hyebeen Kim^1,2^†, Jinwoo Lee^1,3^†, Chang-Lim Yu^1,2^, Chae-Eun Yun^1,2^, Hyeon-Ju Jeong^1,2^, Eun-Ju Jin^1^, Yunju Jo^1^, Dongryeol Ryu^1*^, Gyu-Un Bae^4*^, Jong-Sun Kang^1,2*^

**Supplementary figure legends**

**
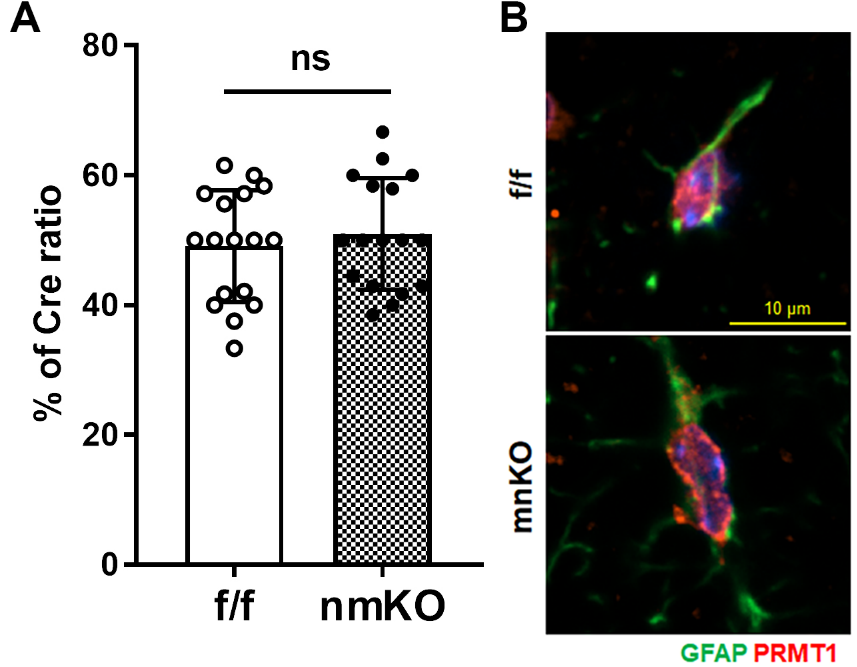
**

**Fig. S1 Motor neuron-specific ablation of Prmt1. (A)** The ratio of f/f and mnKO at postnatal 10 days. Data are expressed as mean ± SD. **(B)** Glial expression of Prmt1 in f/f and mnKO mice. To determine statistical significance, an unpaired two-tailed student t-test was used. n.s = not significant.


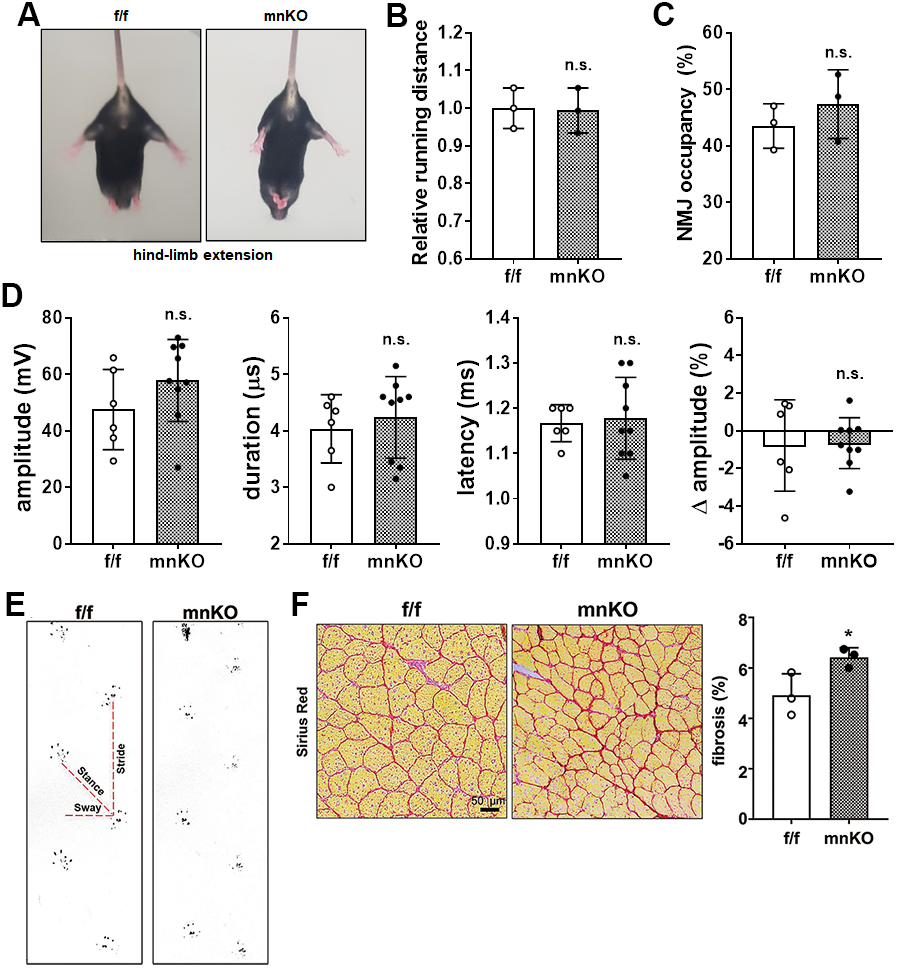


**Fig. S2 mnKO mice exhibit impaired motor function recovery and muscle degradation after post sciatic nerve injury. (A)** Representative images of hind-limb extension of 3 to 5-month-old f/f and mnKO mice. **(B)** Treadmill exercise test of 3-month-old f/f and mnKO mice. **(C)** NMJ occupancy of 3-month-old f/f and mnKO mice. **(D)** Nerve conduction study of 5-month-old f/f (n = 6) and mnKO (n = 9) mice. **(E)** Gait tracing from hindlimb footprint test of f/f and mnKO mice at PID28. **(F)** Sirius red staining for the assessment of fibrosis. Quantification of relative fibrotic area was measured by Image J software. Data are expressed as mean ± SD. To determine statistical significance, an unpaired two-tailed student t-test was used. *p < 0.05.


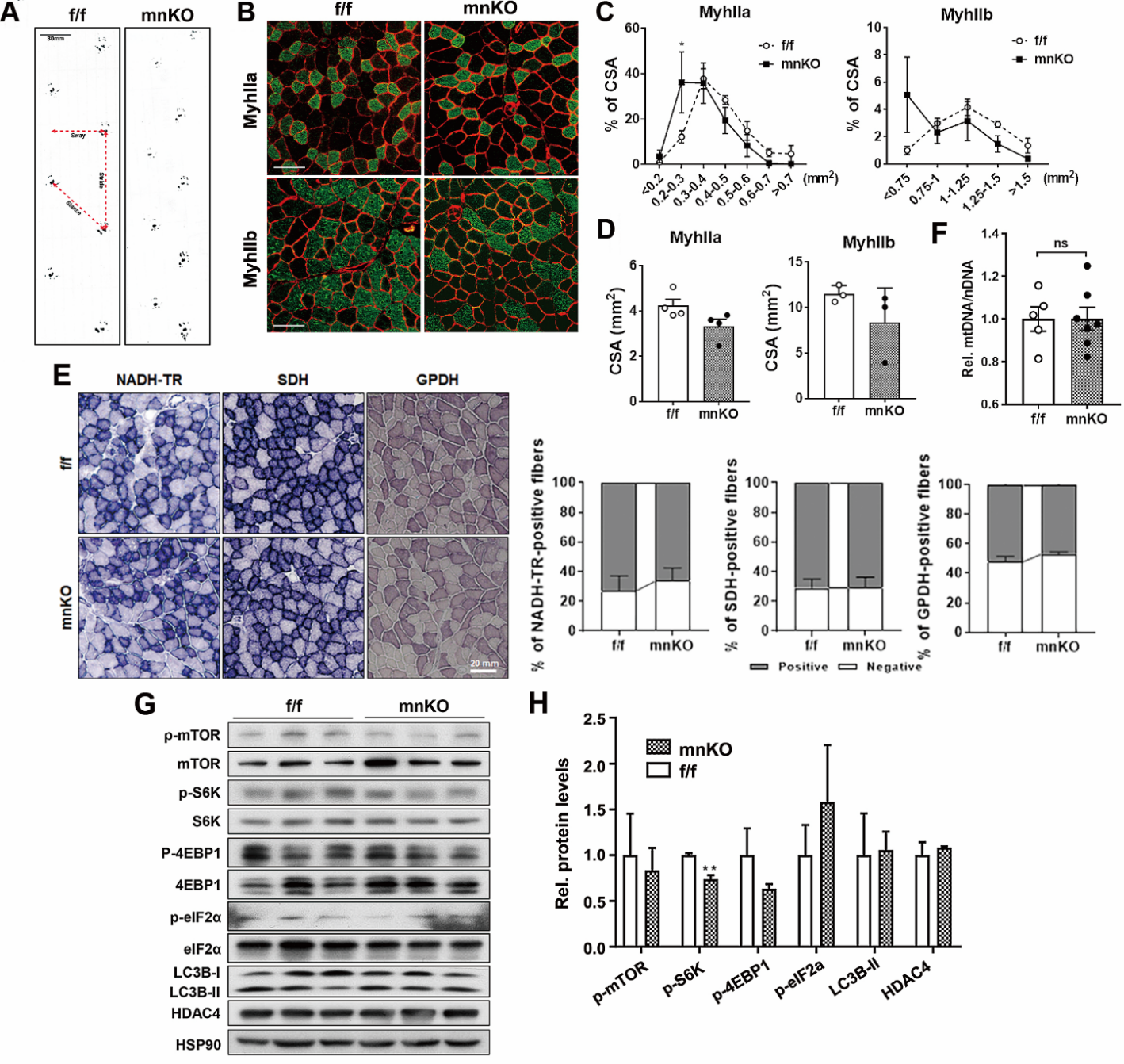


**Fig. S3 Aged mnKO** **mice** **shows impaired muscle function. (A)** Gait tracing from the hindlimb footprint test of aged f/f and mnKO mice. **(B)** Immunostaining for Myosin heavy chain IIa (MHCIIa) and IIb (MHCIIb). Scale bar = 20 μm. **(C)** Proportion of myofibers categorized by the cross-sectional area (CSA). Data are expressed as mean ± SD. **(D)** Average CSA of individual myofiber type. Data are expressed as mean ± SD. **(E)** NADH, SDH, and GPDH staining of f/f and mnKO TA muscles and their quantification. **(F)** The relative mitochondrial DNA (mtDNA)–to–nuclear DNA (nDNA) ratio. Values from the control were set to 1. Data are expressed as mean ± SD. **(G, H)** Immunoblot analysis for p-mTOR, mTOR, p-S6K, S6K, p-4EBP1, 4EBP1, p-eIF2α, eIF2α, LC3B-1 and -II, HDAC4 and HSP09 in muscle from aged f/f and mnKO mice (G) and their quantifications relative to HSP90 (H). To determine statistical significance, an unpaired two-tailed student t-test was used. *p < 0.05, **p < 0.01; ***p < 0.001.


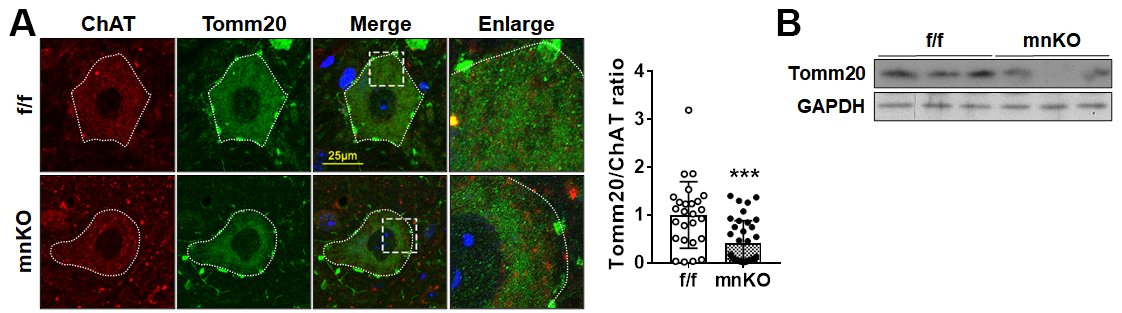


**Fig. S4 Mitochondrial content is decreased in motor neurons of aged mnKO** **mice. (A)** Relative levels of Tomm20 in the f/f and mnKO motor neurons. **(B)** Immunoblot analysis of Tomm20 in lumbar spinal cords of f/f and mnKO mice. GAPDH serves as a loading control. Data are expressed as mean ± SD. To determine statistical significance, an unpaired two-tailed student t-test was used. ***p < 0.001.

**Table S1. Sequences of qRT-PCR primers.**

**
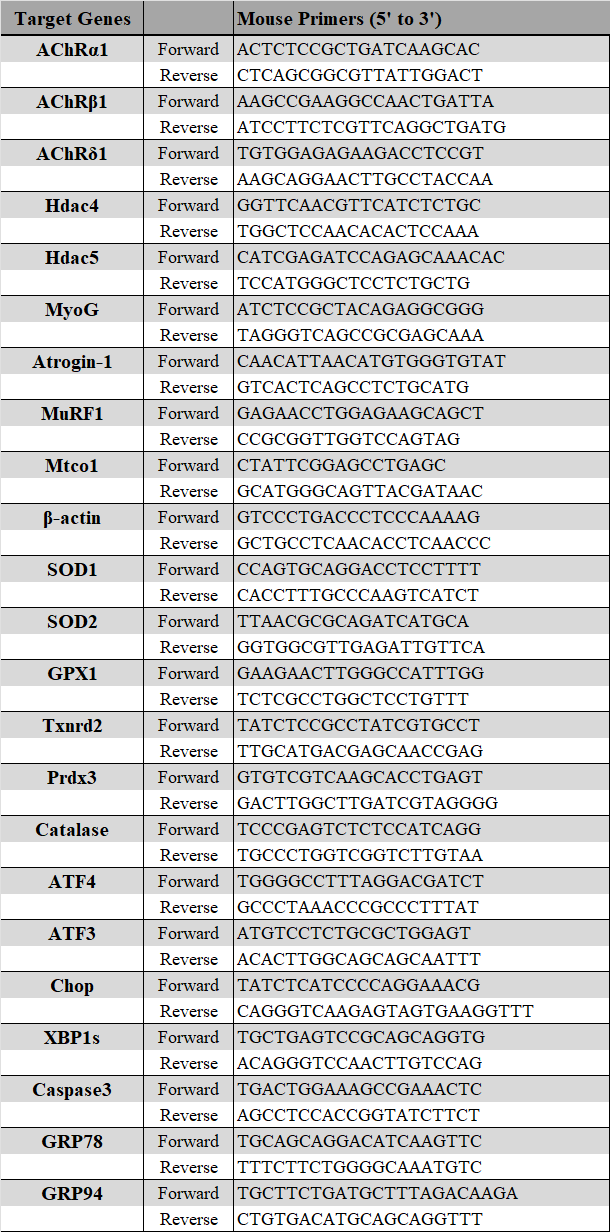
**

**Table S2. Information of immunoblot analysis.**

**
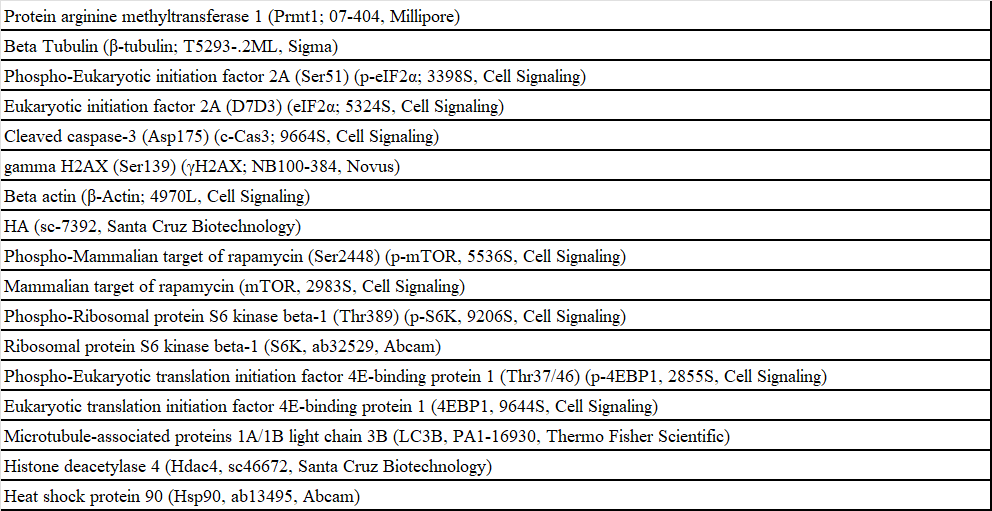
**
